# Aqueous Extract of *Solanum nigrum* Leaves Induces Autophagy and Enhances Cytotoxicity of Cisplatin, Doxorubicin, Docetaxel, and 5-Fluorouracil in Human Colorectal Carcinoma Cells

**DOI:** 10.1155/2013/514719

**Published:** 2013-06-17

**Authors:** Chen-Jei Tai, Chien-Kai Wang, Cheng-Jeng Tai, Yi-Feng Lin, Chi-Shian Lin, Jiun-Yu Jian, Yu-Jia Chang, Chun-Chao Chang

**Affiliations:** ^1^Department of Chinese Medicine, Taipei Medical University Hospital, Taipei 11031, Taiwan; ^2^Department of Obstetrics and Gynecology, School of Medicine, College of Medicine, Taipei Medical University, Taipei 11031, Taiwan; ^3^Division of Hematology and Oncology, Department of Internal Medicine, Taipei Medical University Hospital, Taipei 11031, Taiwan; ^4^Department of Internal Medicine, School of Medicine, College of Medicine, Taipei Medical University, Taipei 11031, Taiwan; ^5^Division of General Surgery, Department of Surgery, Chi Mei Hospital Chiali, Tainan 72263, Taiwan; ^6^Graduate Institute of Medical Sciences, College of Medicine, Taipei Medical University, Taipei 11031, Taiwan; ^7^Graduate Institute of Clinical Medicine, College of Medicine, Taipei Medical University, Taipei 11031, Taiwan; ^8^Department of Surgery, Taipei Medical University and Hospital, Taipei 11031, Taiwan; ^9^Division of General Surgery, Department of Surgery, Taipei Medical University Hospital, Taipei Medical University, Taipei 11031, Taiwan; ^10^Division of Gastroenterology and Hepatology, Department of Internal Medicine, Taipei Medical University Hospital, Taipei 11031, Taiwan

## Abstract

Colorectal cancer is a common cancer worldwide, and chemotherapy is a mainstream approach for advanced and recurrent cases. Development of effective complementary drugs could help improve tumor suppression efficiency and control adverse effects from chemotherapy. The aqueous extract of *Solanum nigrum* leaves (AE-SN) is an essential component in many traditional Chinese medicine formulas for treating cancer, but there is a lack of evidence verifying its tumor suppression efficacy in colorectal cancer. The purpose of this study is to evaluate the tumor suppression efficacy of AE-SN using DLD-1 and HT-29 human colorectal carcinoma cells and examine the combined drug effect when combined with the chemotherapeutic drugs cisplatin, doxorubicin, docetaxel, and 5-fluorouracil. The results indicated that AE-SN induced autophagy via microtubule-associated protein 1 light chain 3 A/B II accumulation but not caspase-3-dependent apoptosis in both cell lines. The IC_50_s after 48 hours of treatment were 0.541 and 0.948 mg/ml AE-SN in DLD-1 and HT-29, respectively. AE-SN also demonstrated a combined drug effect with all tested drugs by enhancing cytotoxicity in tumor cells. Our results suggest that AE-SN has potential in the development of complementary chemotherapy for colorectal cancer.

## 1. Introduction

Colorectal cancer is one of the most common types of cancer worldwide with particularly high incidences in developed countries [1]. In Taiwan, colorectal cancer is already the most common type of cancer and the third most common cause of cancer deaths [2]. Currently, surgery is still the only curative treatment for colorectal cancer. Although 75–80% of newly diagnosed cases are localized or regional tumors, around 50% of patients suffer recurrence after surgery [3, 4]. adjuvant therapy such as postoperative chemotherapy is used to eliminate remaining lesions and help control the risk of recurrence. Chemotherapy is also one of the main treatment approaches in advanced and recurrent cases. However, chemotherapy is often associated with adverse side effects in patients, particularly in the elderly population. Various drug resistance problems in colorectal cancer cases also reduce the response rates. These clinical features limit the performance of chemotherapy in patients. Hence, in order to reduce systematic side effects and improve the tumor suppression capability of chemotherapy, the use of complementary and alternative medicine has become increasingly popular during the last few decades, particularly in Western countries [5–7]. Any effective drug which promotes the tumor suppression efficacy of chemotherapeutic regimens or eases the associated adverse effects may serve as an appropriate candidate to establish an integrated chemotherapy and improve clinical outcomes in cancer patients.

One approach in developing integrated chemotherapy is to choose a drug which enhances tumor cell suppression efficiency by increasing cytotoxicity using a different cell death mechanism from other drugs used in the regimen. In general, the tumor suppression mechanisms of current chemotherapeutic drugs are mainly based on disruption of cell cycle processes, resulting in cell apoptosis. For example, well-studied chemotherapeutic drugs such as the alkylating agents, cisplatin, and carboplatin inhibit DNA synthesis in tumor cell growth by forming DNA adducts [8, 9], whereas plant alkaloids, such as the taxanes, block tumor cell mitosis by stabilizing tubulin [10, 11]. Other common drugs such as doxorubicin function as topoisomerase II inhibitors and interfere with DNA/RNA synthesis in tumor cells [12, 13]. The currently recommended chemotherapeutic drug for colorectal cancer is 5-fluorouracil (5-Fu) [3, 14–16], which activates apoptosis by incorporation of DNA/RNA on cancer cells [17]. Treatments using these common chemotherapeutic drugs normally lead to cell cycle arrest and activate the apoptotic process in tumor cells. Combining standard chemotherapeutics with antitumor drugs to induce tumor cell death via other molecular pathways would not only improve tumor suppression efficiency but also reduce the doses of chemotherapeutic drugs, which could help control adverse effects and may slow the development of drug resistance. 

Traditional Chinese medicine (TCM) is based on the use of natural products and well-established theoretical approaches. TCM provides many potential candidates as effective drugs for integrated cancer chemotherapy, such as TJ-41 (Bu-Zhong-Yi-Qi-Tang) and PHY906 (Huang-Qin-Tang) [18–20]. In TCM practice, a therapeutic formula is normally prepared as an aqueous extract mixed with various medical herbs. One major herb in this formula is responsible for relieving the target symptom, whereas other medicinal herbs are added to enhance the therapeutic effects or reduce the side effects of the major herb. *Solanum nigrum* (SN) is frequently used as an elemental ingredient for clinical TCM cancer therapy [8]. Recently, many *in vitro* studies have demonstrated the antitumor effects of SN extracts on various cancer types, including leukemia and prostate, liver, breast, lung, stomach, colon, bladder, and endometrial cancers [8, 21–24]. In these studies, the SN-related antitumor effect was thought to occur via activation of apoptosis and autophagy in human cancer cells, particularly when using the aqueous extract of SN (AE-SN) [22, 25]. However, most studies on the treatment effect on colon cancer have mainly assessed the tumor suppression capability of pure compounds such as solamargines and degalactotigonin [21], rather than evaluating the antitumor efficiency and mechanism of AE-SN. The tumor suppression efficacy of AE-SN in colon cancer cells therefore remains unclear. We previously observed that AE-SN has a combined drug effect with the standard chemotherapeutic drug docetaxel in human endometrial cancer cells [24]. This observation suggests that AE-SN may also enhance cytotoxicity of chemotherapeutic drugs in human colorectal cancer cells.

The aim of the present study is to evaluate the tumor suppression efficacy of AE-SN alone and combined drug effects of AE-SN with the common chemotherapeutic drugs cisplatin, doxorubicin, and 5-Fu docetaxel on human colorectal cells. This information could be helpful in improving the tumor suppression efficiency of chemotherapy for colorectal cancer. 

## 2. Materials and Methods

### 2.1. Plant Materials and Preparation of AE-SN

In TCM practice, the drug form of SN is generally prepared as the aqueous extract of SN leaves (AE-SN). The preparation of AE-SN for the present study was therefore based on the TCM processing method. Briefly, 50 g of the dried leaf part of *Solanum nigrum* was immersed in 750 mL distilled water. This raw solution was gradually heated to 100°C within 50 min and maintained at 100°C for one hour. This AE-SN solution was further concentrated to 1 g/mL. 

### 2.2. Cell Culture. 

The human colorectal carcinoma cell lines, HT-29 and DLD-1, were a gift from Dr Pei-Yi Tsai (Department of Animal Pharmacology, Development Center for Biotechnology, Taipei, Taiwan) and purchased from the Bioresource Collection and Research Center (Hsinchu, Taiwan), respectively. Both HT-29 and DLD-1 cells contain a p53 mutation. Cells were cultured in Dulbecco's modified Eagle's medium/nutrient mixture F-12 medium (Gibco, Grand Island, NY, USA) with 100 U/mL of penicillin and 100 *μ*g/mL streptomycin (Invitrogen Life Technologies, Carlsbad, CA, USA) at 37°C in a 5% CO_2_ humidified incubator. 

### 2.3. Cytotoxicity Assay and Microscopic Observation

 HT-29 cells or DLD-1 cells were seeded into 96-well microplates at a density of 5 × 10^3^ cells per well overnight and then treated with 0, 0.05, 0.1, 0.2, 0.5, 1, 2, and 5 mg/mL AE-SN for 24 or 48 hr. In order to clarify the autophagic cell death on AE-SN treated cells, three autophagy inhibitors, 3-methyladenine (3-MA), bafilomycin A, or pepstatin A/E64d (Sigma-Aldrich, St Louis, MO, USA), were treated with AE-SN on HT-29 and DLD-1 cells. The cytotoxicity of AE-SN on tumor cells was then determined by 3-(4,5-dimethylthiazol-2-yl)-2, 5-diphenyltetrazolium bromide (MTT) assay. A trypan blue exclusion test was also performed to confirm the cell viability determined by MTT assay. HT-29 cells or DLD-1 cells were seeded into 24-well plates at a density of 3 × 10^4^ cells per well overnight and then treated with 0, 0.2, 0.5, or 1 mg/mL AE-SN. After 48 hr incubation, cells were harvested by trypsinization and centrifuged at 100 ×g for 5 min to collect the cell pellet which was then resuspended in prewarmed phosphate-buffered saline with trypan blue (Sigma-Aldrich, St Louis, MO, USA) at a 1 : 1 ratio for 3 min at room temperature. The number of live cells without trypan blue staining was counted by two independent observers using a hemocytometer under a microscope. In the study of AE-SN combined with cisplatin, doxorubicin, and docetaxel, cells were treated with a series of cisplatin, doxorubicin, or docetaxel with 0, 0.5, or 1 mg/mL AE-SN for 48 hr. Cisplatin, doxorubicin and 5-fluorouracil (5-Fu) were purchased from Sigma-Aldrich (St. Louis, MO, USA) and docetaxel (Tyxan) from TTY Biopharm (Taipei, Taiwan). Cell morphology was observed with a Nikon Eclipse TS100 optical microscope (Nikon Instruments, Melville, NY, USA) and photographed at 100x magnification. Cytotoxicity was also determined by MTT assay.

### 2.4. Western Blotting Analysis of Cell Death Markers

HT-29 and DLD-1 cells (5 × 10^5^ cells per dish) were seeded in 6 cm dishes overnight and incubated with 0 or 1 mg/mL AE-SN alone, or in combination with cisplatin, doxorubicin, or docetaxel for 48 hr. Cells were harvested by RIPA buffer (150 mM NaCl, 50 mM pH 7.5 Tris-HCL, 1% NP-40, 0.5% deoxycholate, 0.1% SDS, 1 mM PMSF, 10 *μ*g/mL leupeptin and 100 *μ*g/mL aprotinin). The total protein concentration of the cell extracts was determined by a Bio-Rad protein assay kit (Bio-Rad Laboratories, Hercules, CA, USA). Each cell extract was then equalized to 30 *μ*g and separated using 12% sodium dodecyl sulfate polyacrylamide gel electrophoresis. The proteins were transferred onto a polyvinylidene fluoride membrane (Pall Corp, Port Washington, NY, USA) and probed with the primary antibodies, caspase-3 (1 : 1,000), mammalian microtubule-associated protein 1 light chain 3 A/B (LC3A/B, 1 : 1,000), and glyceraldehyde 3-phosphate dehydrogenase (GAPDH, 1 : 10,000), followed by donkey anti-rabbit horseradish peroxidase-conjugated secondary antibody (1 : 10,000, Santa Cruz Biotechnology, Santa Cruz, CA, USA). This anti-LC3 A/B identifies the LC3 A/B I and II forms with molecular masses of 16 and 14 kDa, respectively. The accumulation of LC3 A/B II forms is considered a protein marker for activation of autophagy [26], whereas the anticaspase-3 recognizes procasapse-3 3 with a mass of 35 kDa and cleaved caspase-3 with masses of 19 and 17 kDa. Anti-caspase-3 and LC3 A/B were purchased from Cell Signaling Technology (Danvers, MA, USA), and anti-GAPDH was purchased from Abfroniter (Seoul, Republic of Korea). Immunoreactivity was then detected with a WesternBright electrochemiluminescence Western blotting detection kit (Advabsta, Menlo Park, CA, USA). Semiquantitative analysis of the intensity of the immunoreactive bands was performed by ImageJ software (National Institutes of Health, Bethesda, MD, USA).

### 2.5. Statistical Analysis

Data from cell viability and semiquantitative Western blotting analysis were presented as mean ± stand and derivation (SD). Statistical significance was analyzed by Student's *t*-test when comparing two different groups and one-way ANOVA when examining the dose-dependent effect. Statistical analysis was performed by SPSS (SPSS Inc, Chicago, IL, USA). 

CalcuSyn software (Biosoft, Cambridge, UK) was used for the statistical analysis of the half maximal inhibitory concentration (IC_50_) of AE-SN determined by MTT assay and for the combined effects of AE-SN with chemotherapeutic drugs. Statistical analysis of the combined drug effects with the CalcuSyn software is based on the median-effect method and evaluated by the combination index (CI) value; a synergistic effect of two drugs was determined when the calculated CI value was less than 1 [27].

## 3. Results

### 3.1. AE-SN Cytoxicity and Induction of Autophagy in HT-29 and DLD-1 Human Colorectal Carcinoma Cells

**Figure 1:**
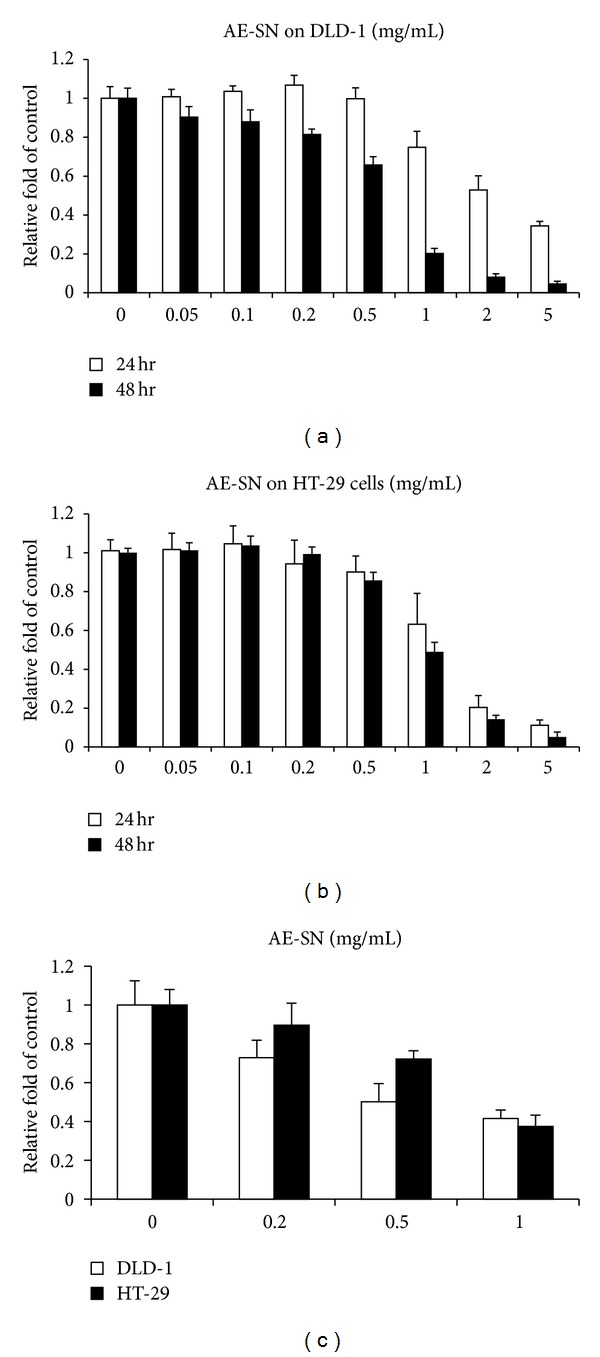
Cytotoxicity of AE-SN in DLD-1 and HT-29 human colorectal carcinoma cells (a); DLD-1 cells were treated with 0.05 to 5 mg/mL AE-SN for 24 or 48 hr; (b), HT-29 cells were treated with 0.05 to 5 mg/mL AE-SN for 24 hr; the cytotoxicity of (a) and (b) was analyzed by MTT assay; (c)DLD-1 or HT-29 cells were treated with 0.2 to 1 mg/mL AE-SN; and the cytotoxicity was analyzed by a trypan blue exclusion test. Experiments were performed in triplicate and data are shown as mean ± SD. Both cell lines showed dose-dependent effects with AE-SN treatment at 24 and 48 hr in the MTT assay and trypan blue exclusion test (one-way ANOVA, *P* < 0.001). AE-SN: aqueous extract of *Solanum nigrum*: MTT: 3-(4,5-dimethylthiazol-2-yl)-2,5-diphenyltetrazolium bromide.

**Figure 2:**
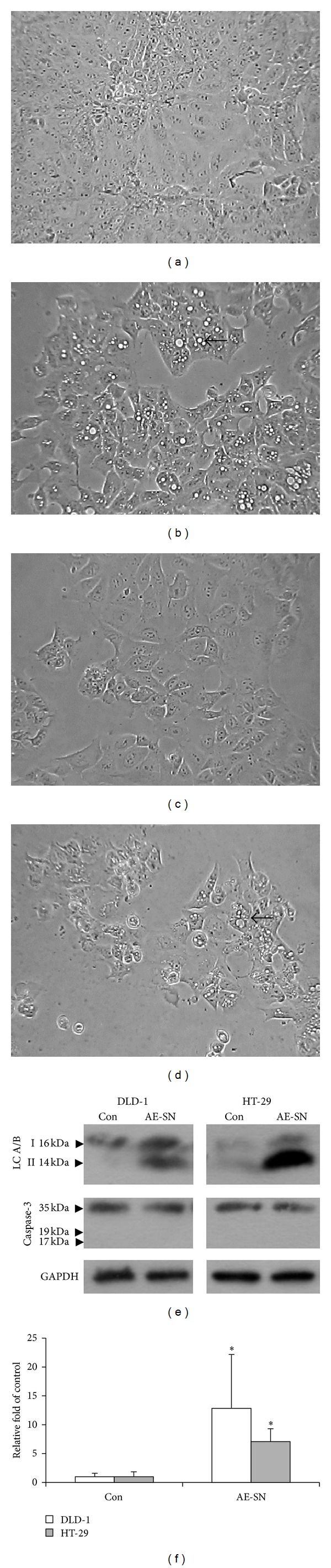
AE-SN induced morphological changes and LC-3 activation in DLD-1 and HT-29 cells. (a) and (b), DLD-1 cells were treated with 0 or 1 mg/mL AE-SN for 48 hr, respectively; (c) and (d), HT-29 cells were treated with 0 or 1 mg/mL AE-SN for 48 hr. Arrows indicate lipid droplet-like morphological changes in AE-SN-treated cells. Magnification = 100x. (e) Total protein extracts were harvested from DLD-1 cells or HT-29 cells which were treated with 0 or 1 mg/mL AE-SN (control versus AE-SN) for 48 hr. Activation of caspase-3 and LC3 A/B in AE-SN-treated cells was determined by western blotting analysis. (f) Semiquantification of LC-3 A/B II in AE-SN-treated cells. Data presented are mean ± SD (*n* = 5). indicates statistical significance compared with 0 mg/mL AE-SN treatment using Student's *t*-test (*P* < 0.05). AE-SN: aqueous extract of *Solanum nigrum*; Con: control; GADPH: glyceraldehyde 3-phosphate dehydrogenase; LC-3 A/B II: mammalian microtubule-associated protein 1 light chain 3 A/B II.

As shown in Figure 1, AE-SN induced cytotoxicity in both DLD-1 and HT-29 cells in a dose-dependent manner. According to the cytotoxicity results obtained from MTT assay, the IC_50_s after 48 hr AE-SN treatment were 0.541 and 0.948 mg/mL AE-SN in DLD-1 and HT-29 cells, respectively (Table 1). In contrast, the trypan blue exclusive test indicated that the cell viability of DLD-1 under 0.5 mg/mL AE-SN treatment was 50.1%, whereas that of HT-29 under 1.0 mg/mL AE-SN treatment was 37.4%. These conflicting results indicated that the MTT assay may underestimate the real cytotoxicity of AE-SN because of disruption of mitochondrial metabolism. Under microscopic inspection, lipid-like droplets were observed in AE-SN-treated DLD-1 and HT-29 cells (Figures 2(a)–2(d)). This morphological feature suggested that AE-SN-treated cells were in the autophagic process which may be related to AE-SN-induced cytotoxicity. On the other hand, no apoptosis-related morphological changes such as cell shrinkage or chromatin condensation were observed in AE-SN-treated colorectal carcinoma cells. To further examine the cell death mechanisms involved in AE-SN-induced cytotoxicity, the activation of caspase-3, the protein marker for apoptosis, and the accumulation of LC3 A/B II, the protein marker for autophagy, were determined by western blotting analysis. Figures 2(e) and 2(f) demonstrate that accumulation of LC3 A/B II was significantly increased in AE-SN-treated colorectal carcinoma cells, whereas activation of caspase-3 was not observed. The accumulation of LC3 A/B II is a protein marker which identifies autophagy in cells, and Western blotting analysis indicated that AE-SN treatment increased LC3 A/B II accumulation 12.83- and 7.08- fold in DLD-1 and HT-29 cells, respectively (Figure 2(e)). These results suggested that AE-SN was effective in suppressing tumor cell growth in DLD-1 and HT-29 human colorectal carcinoma cells and induced accumulation of LC3 A/B II and the autophagic process, but not caspase-3-dependent apoptosis.

**Figure 3:**
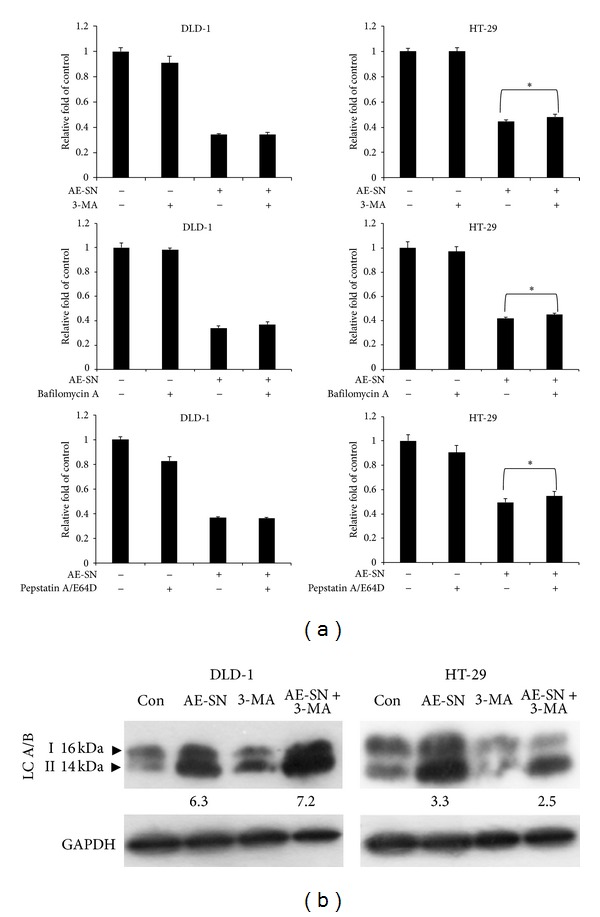
Inhibition of AE-SN induced autophagy by using 3-MA, bafilomycin (a), and pepstatin A/E64d on DLD-1 and HT-29 cells. (a) DLD-1 and HT-29 cells were treated with 0.5 or 1.0 mg/mL AE-SN, respectively, in combination with 1 *μ*M 3-MA, 2 nM bafilomycin A, or 2 *μ*g/mL pepstatin A/E64d for 48 hr. Cytotoxicity was analyzed by MTT assay. Experiments were performed in triplicate and the data shown are mean ± SD. *indicates statistical significance compared with 0 mg/mL AE-SN treatment using Student's *t*-test (*P* < 0.05). (b) DLD-1 and HT-29 cells were treated with control, AE-SN (1 mg/mL), 3-MA, or AE-SN plus 3-MA (*μ*M) for 48 hr and accumtrluation of LC3 A/B II was determined by Western blotting. Numbers indicated fold induction of LC3 A/B II compared with control. AE-SN: aqueous extract of *Solanum nigrum*; MTT: 3-(4,5-dimethylthiazol-2-yl)-2, 5-diphenyltetrazolium bromide.

To clarify the role of AE-SN-activated autophagy on HT-29 and DLD-1 cells, AE-SN-treated HT-29 and DLD-1 cells were also cotreated with autophagic inhibitors: 3-MA, bafilomycin A, or pepstatin A/E64d. 3-MA is a class III phosphoinositide-3 kinase inhibitor to block LC3 A/B I to II conversion [28], bafilomycin A is a lysosomal inhibitor to prevent the fusion of lysosomes and autophagosomes [29], and pepstatin A/E64d is a cathepsins inhibitor to interfere with autolysosomal digestion [30]. As shown in Figures 3(a), slightly recovery of cell viability was observed on AE-SN and autophagic inhibitors cotreated HT-29 cells, whereas no difference was observed in DLD-1 cells. Cotreatment of AE-SN and 3-MA only reduced AE-SN-induced accumulation of LC-3 A/B II from 3.33- to 2.5-fold of control in HT-29 cells (Figure 3(b)). These results suggested that autophagy was partly involved in AE-SN-induced cell death in HT-29, but not DLD-1 cells.

### 3.2. Combined Drug Effects of AE-SN and Cisplatin, Doxorubicin, Docetaxel, and 5-Fu

**Figure 4:**
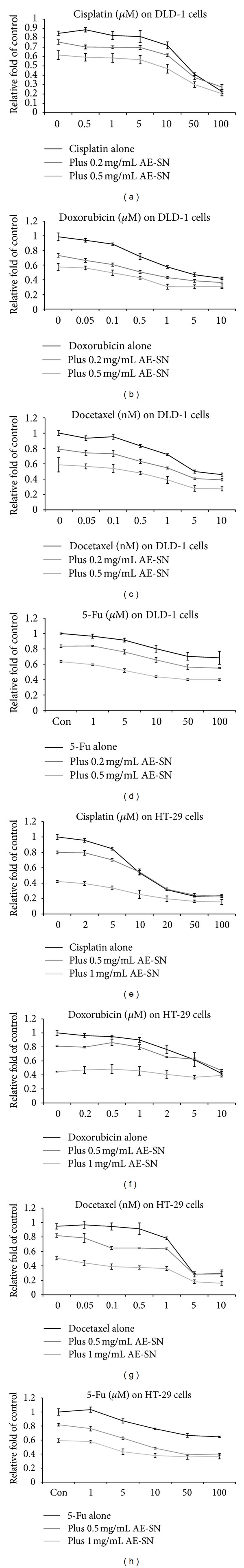
Cytotoxicity of a combination of AE-SN and the chemotherapeutic drugs, cisplatin, doxorubicin, docetaxel, or 5-Fu. (a), (b), (c), and (d), DLD-1 cells were treated with 0 to 100 *μ*M cisplatin, 0 to 10*μ*M doxorubicin, 0 to 10 nM docetaxel, 0 to 100 *μ*M 5-Fu in combination with 0, 0.2 or 5 mg/mL AE-SN for 48 hr; (e), (f), (g), and (h) HT-29 cells were treated with 0 to 100 *μ*M cisplatin, 0 to 10*μ*M doxorubicin, and 0 to 10 nM docetaxel, or 0 to 100 *μ*M 5-Fu in combination with 0, 0.5 or 1 mg/mL AE-SN for 48 hr. Cytotoxicity was analyzed by MTT assay. Experiments were performed in triplicate and the data shown are mean ± SD. AE-SN: aqueous extract of *Solanum nigrum*; MTT: 3-(4,5-dimethylthiazol-2-yl)-2, 5-diphenyltetrazolium bromide.

Since AE-SN effectively induced caspase-3- independent cell death in DLD-1 and HT-29 cells, the present study further examined the potential of AE-SN in combination with chemotherapeutic drugs which are associated with apoptotic cell death. Tumor cells were treated by serial doses of four chemotherapeutic drugs, cisplatin, doxorubicin, docetaxel or 5-fu alone or in combination with AE-SN for 48 hr. According to previous results, HT-29 cells are less sensitive to AE-SN and therefore the selected AE-SN doses for combination treatment were 0.5 and 1.0 mg/mL, whereas doses of 0.2 and 0.5 mg/mL were chosen for DLD-1 cells. Cytotoxicity was determined by MTT assay and the combined drug effect was further analyzed. AE-SN presented an enhanced cytotoxicity in combination with all four chemotherapeutic drugs in both cell lines (Figure 4). Analysis showed the synergy of AE-SN combined with each chemotherapeutic drug (CI less than 1) (Table 1). The IC_50_ of each regimen also significantly decreased in AE-SN-combined treatment in comparison with each chemotherapeutic drug alone (Table 1). Together, these *in vitro *results suggest that AE-SN can potentially enhance the tumor suppression effect in human colorectal carcinoma cells when combined with cisplatin, doxorubicin, docetaxel and, 5-Fu.

### 3.3. Activation of LC3 A/B and Caspase-3 in AE-SN and Chemotherapeutic Drug-Treated Cancer Cells

**Figure 5:**
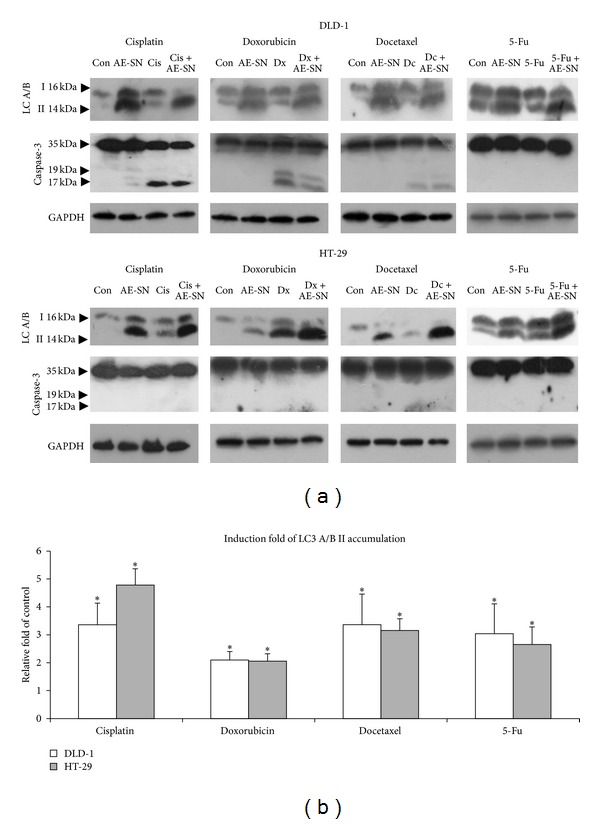
Activation of cell death markers, LC3 A/B and caspase-3 in DLD-1 and HT-29 cells treated with AE-SN and cisplatin, doxorubicin, or docetaxel. Both DLD-1 and HT-29 cells were treated with 0, 1 mg/mL AE-SN, chemotherapeutic drugs alone, and a combination of 1 mg/mL AE-SN with chemotherapeutic drugs. The doses of cisplatin used were 50 *μ*M in DLD-1 and 20 *μ*M in HT-29. The dose of doxorubicin used was 5*μ*M in DLD-1 and HT-29. The dose of docetaxel used was 1 nM in DLD-1 and HT-29, whereas the dose of 5-Fu was 20*μ*M. After 48 hr incubation, total protein extracts were harvested from cells. (a) Activation of LC3 A/B and caspase-3 in DLD-1 and HT-29 cells was determined by western blotting analysis; (b) semiquantification of LC3 A/B II is presented as the relative fold induction of the control as mean ± SD (*n* = 3). *indicates statistical significance compared with 0 mg/mL AE-SN treatment using Student's *t*-test (*P* < 0.05). AE-SN: aqueous extract of *Solanum nigrum*; GADPH: glyceraldehyde 3-phosphate dehydrogenase; LC-3 A/B II: mammalian microtubule-associated protein 1 light chain 3 A/B II.

The four tested chemotherapeutic drugs are well known to induce apoptosis via activation of caspase-3 in many tumor cells. This raised a possibility that AE-SN-enhanced cytotoxicity is due to a combined effect of AE-SN-induced caspase-3- independent cell death and chemotherapeutic drug-induced apoptotic cell death. In order to examine this hypothesis, DLD-1 cells or HT-29 cells were treated with 0 or 1 mg/mL AE-SN in combination with cisplatin (50 or 20 *μ*M), doxorubicin (5 *μ*M for both), docetaxel (1 nM for both), and 5-Fu (50 *μ*M) for 48 hr and total protein extracts were harvested. The apoptotic protein marker, cleaved caspase-3, and autophagic protein marker, LC3 A/B II, were then determined in the total protein extracts of the cells. As shown in Figure 5(a), all tested drugs activated caspase-3 and produced cleaved caspase-3 in DLD-1, but not HT-29 cells, whereas AE-SN increased LC3 A/B II in both cell lines. Semiquantitative data further confirmed that AE-SN treatment induced LC3 A/B II accumulation in chemotherapeutic drug-treated cells (Figure 5(b)). In cisplatin-treated cells, cotreatment with 1 mg/mL AE-SN increased LC3 A/B II 3.35- and 4.78-fold, whereas in docetaxel treatment, AE-SN also increased the autophagic marker by 3.36- and 3.15-fold in DLD-1 and HT-29 cells, respectively. In combination with 5-Fu, AE-SN induced 3.03- and 2.65-fold of LC3 A/B II accumulation, similar to docetaxel. In doxorubicin-treated DLD-1 and HT-29 cells, the fold induction of LC3 A/B II was 2.09- and 2.05-fold, respectively. It is slightly lower than cisplatin, docetaxel, and 5-Fu. When both cell lines were exposed to a combination of AE-SN and the test drugs, activation of caspase-3 and LC3 A/B II were observed together (Figure 5(a)). This result suggested that the AE-SN enhanced cytotoxicity with chemotherapeutic drugs and also induced LC3 A/B II accumulation in colorectal carcinoma cells.

## 4. Discussion

The tumor suppression efficacy and related mechanisms of AE-SN have been examined in some cancer types. For instance, AE-SN induced both cleavage of caspase-3 and accumulation of LC3 A/B II in HepG2 human hepatocellular carcinoma cells [25], and similar results were observed in AU565 human breast carcinoma cells [22]. Interestingly, AE-SN treatment only induced LC3 A/B II accumulation but not cleavage of caspase-3in HEC-1A, HEC-1B, and KLE human endometrial cells [24], with similar results demonstrated in DLD-1 and HT-29, human colorectal carcinoma cells. AE-SN-induced autophagy was suggested to cause cancer cell death, particularly in human hepatocellular carcinoma cells, HepG2 [25], and breast adenocarcinoma cells, AU565 [22]. However, autophagic cell death was only identified in HepG2 cells by using 3-MA, a phosphoinositide 3-kinase inhibitor [25]. In cancer cells, autophagy plays a dual role in performing cytoprotection or programmed cell death (reviewed by [31, 32]. For example, autophagy protected colorectal cancer cells HCT-116 and DLD-1 from 5-Fu-induced apoptosis [33]. In present study, autophagic inhibitors such as 3-MA, bafilomycin, and pepstatin A/E64d only slightly recovered cell viability and reduced accumulation of LC3 A/B II on HT-29 cells. These results indicated that AE-SN activated autophagy may partly contribute to AE-SN-induced cell death on HT-29 cells, but not DLD-1 cells. On the other hand, treatment of autophagic inhibitors cannot further promote cell death suggesting that AE-SN-induced autophagy may not be involved in cytoprotective effect on HT-29 and DLD-1 cells. Since both caspase-3- dependent apoptosis and autophagy were not fully responsible for AE-SN-induced cell death on HT-29 and DLD-1 cells, the exact cell death mechanism activated by AE-SN still remained unclear. 

In the present study, AE-SN demonstrated an effective cytotoxicity in human colorectal carcinoma cells. Both DLD-1 and HT-29 cell lines have p53 mutations and are partially resistant to the p53-mediated apoptotic pathway [34, 35]. AE-SN may therefore fail to activate apoptosis via p53 but may still be capable of activating cell death in both cell lines. Lee and colleagues suggested that SN-isolated glycoprotein induced caspase-3-dependent apoptosis in HT-29 cells [36], whereas the present results indicate that AE-SN treatment cannot activate cleavage of caspase-3 in HT-29 cells. These conflicting results provide an important reminder that the biological effects of medicinal herbs may vary according to different preparation and purification methods. Moreover, several studies suggested that the AE-SN-induced apoptosis is due to the presence of steroid alkaloids and phytoglycoproteins in AE-SN [21, 37–39], whereas the chemical compound responsible for AE-SN-induced tumor cell death remained unknown. In order to clarify the exact tumor suppression mechanism, further investigation for identification of effective compounds in AE-SN is therefore required. Nevertheless, the present data suggest that AE-SN may be a potential drug for dealing with colorectal cancer cells, particularly when they are resistant to apoptosis-based chemotherapeutic drugs. 

Since AE-SNinduced cytotoxicity in a caspase-3- independent manner, cotreatment of AE-SN with apoptotic activators may further enhance cytotoxicity in tumor cells. AE-SN co-treatment significantly enhanced cytotoxicity in all tested chemotherapy drugs. Although HT-29 is a more AE-SN-resistant cell line, a low dose of AE-SN at 0.5 mg/mL still demonstrated a synergistic effect as good as a higher dose of 1 mg/mL with all tested drugs. AE-SN-induced LC3 A/B II accumulation was also present in cisplatin/doxorubicin/docetaxel-treated cells, and cleaved caspase-3 was induced by these chemotherapeutic drugs in DLD-1, but not HT-29 cells. Meanwhile, 5-Fu treatment induced cell death independent of activation of caspase-3- in both cell lines. Both caspase-3 dependent/independent cell death induced by chemotherapeutic drugs can be further enhanced by AE-SN treatment. Compared with AE-SN treatment alone, co-treatment with chemotherapeutic drugs decreased the fold induction of LC3 A/B II in both cell lines. For example, cotreatment of 1 mg/mL AE-SN and 5 *μ*M doxorubicin decreased the induction fold from 12.83 and 7.08-fold to 2.09 and 2.05-fold in DLD-1 and HT-29 cells, respectively. Moreover, higher fold induction of LC3 A/B II accumulation was observed in HT-29 compared with that absaved in DLD-1 cells (3.35- versus 4.78-fold) with co-treatment of cisplatin and AE-SN. This suggests that treatment with chemotherapeutic drugs may disrupt AE-SN-induced autophagy in colorectal carcinoma cells regardless of the synergistic cytotoxic effects observed under AE-SN co-treatment, and this disruption may differ by cancer cell types and selected chemotherapeutic drugs. Interestingly, autophagy activated by AE-SN on DLD-1 and HT-29 cells had no protective effect against all tested chemotherapeutic drugs. This finding supported the previous study using autophagic inhibitors, and suggested AE-SN induced autophagy did not play a protective role in HT-29 and DLD-1 cells. Further investigation on the relationship of AE-SN-induced autophagy and cell death may help clarify the molecular mechanism involved in the drug cross-interaction. Collectively, these results indicate that AE-SN is a potential candidate for integration in chemotherapeutic regimens. 

Although AE-SN demonstrated an observed tumor suppression efficacy alone and was also capable of enhancing tumor cell death induced by chemotherapeutic drugs the *in vitro* evidence provided here requires further verification *in vivo*. In order to assess the tumor suppression efficiency *in vivo*, the optimal administration approach and dosage of AE-SN alone or in combination with individual chemotherapeutic drugs must be further examined with appropriate animal cancer models. Any unexpected adverse effects of the application of AE-SN must be carefully investigated *in vivo* prior to consideration for further clinical trials. AE-SN dramatically reduced the IC_50_ doses of all tested chemotherapeutic drugs. Reduced dosage of chemotherapeutic drugs also suggests potentially more tolerance of adverse effects by colorectal cancer patients.

## 5. Conclusion

**Figure 6:**
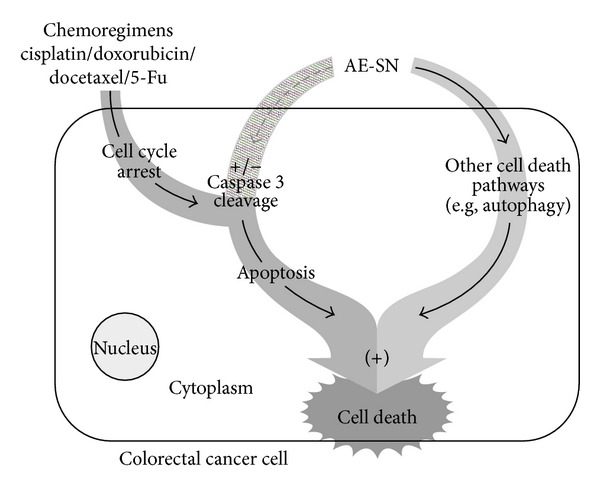
Proposed mechanism for the programmed cell death activated by AE-SN and chemotherapeutic drugs in human colorectal carcinoma cells. AE-SN-activated cell death can increase with cisplatin/doxorubicin/docetaxel/5-Fu-activated caspase-3-dependent or independent cell death (+/− caspase-3 cleavage) and result in further enhanced tumor cell death. (+) indicates synergistic effect of AE-SN with chemotherapeutic drugs. Solid line: activated pathway; the dotted line: inactive pathway. AE-SN: aqueous extract of *Solanum nigrum*.

In this study, AE-SN demonstrated a tumor suppression efficacy against DLD-1 and HT-29 human colorectal carcinoma cells and further enhanced tumor cell death with tested chemotherapeutic drugs in human colorectal carcinoma cells. AE-SN induced tumor cell death in a caspase-3- independent manner on HT-29 and DLD-1 cells with activation of autophagy. Although the full mechanism of AE-SN-induced cell death was still unclear, AE-SN was capable of enhancing cell death activated by cisplatin/doxorubicin/docetaxel/5-Fu (Figure 6). In conclusion, AE-SN is a potential candidate to development of the complementary for colorectal cancer.

## Figures and Tables

**Table 1:** IC_50_ of AE-SN alone and in combination with cisplatin, doxorubicin, docetaxel, or 5-Fu in DLD-1 and HT-29 cells.

			DLD-1						HT-29			
				AE-SN						AE-SN		
			0.2 mg/ml		0.5 mg/ml				0.5 mg/ml		1.0 mg/ml	
Regimens	Dose	IC_50_^a^	IC_50_^b^	CI	IC_50_^b^	CI	Dose	IC_50_^a^	IC_50_^b^	CI	IC_50_^b^	CI
AE-SN (mg/mL)		0.541						0.948				

Cisplatin (*μ*M)		35.565	14.682		0.354			19.032	2.533		0.545	
	0.5			0.207		4.582	2			0.604		0.611
	1			0.205		4.108	5			0.689		0.414
	5			0.248		3.511	10			0.669		0.288
	10			0.129		1.207	20			0.561		0.34
	50			0.063		0.426	50			0.986		0.643
	100			0.053		0.281	100			1.844		1.267

Doxorubicin (*μ*M)		3.556	0.621		0.091			11.863	7.945		0.131	
	0.05			7.221		17.851	0.2			1.012		2.838
	0.1			2.576		4.91	0.5			3.033		4.382
	0.5			0.592		1.394	1			1.473		1.904
	1			0.293		0.165	2			0.679		0.428
	5			0.674		0.429	5			1.21		0.429
	10			1.081		0.822	10			1.097		0.981

Docetaxel (nM)		6.166	2.041		1.887			4.127	1.186		0.021	
	0.05			3.791		7.585	0.05			16.083		18.362
	0.1			3.216		5.008	0.1			2.336		7.342
	0.5			0.888		2.005	0.5			2.577		6.031
	1			0.441		0.545	1			2.564		4.714
	5			0.479		0.235	5			0.331		0.211
	10			0.825		0.397	10			0.729		0.245

5-Fu		NA^c^	NA^c^		6.131			NA^c^	18.51		2.747	
	1			1.229		1.409				1.464		2.747
	5			1.446		1.265				1.202		0.537
	10			0.083		0.429				0.479		0.316
	50			1.139		0.948				0.84		1.018
	100			1.987		1.869				1.85		2.376

^a^Cells treated by AE-SN alone; ^b^cells treated by a combination of AE-SN and the respective chemotherapeutic drug.

AE-SN: aqueous extract of *Solanum nigrum; *IC_50_: half maximal inhibitory concentration.

CI < 1 = synergistic effect.

^c^NA: data not available.
